# Red Cell Distribution Width to Platelet Ratio Is Associated with Increasing In-Hospital Mortality in Critically Ill Patients with Acute Kidney Injury

**DOI:** 10.1155/2022/4802702

**Published:** 2022-01-17

**Authors:** Jiayuan Wu, Liutao Huang, Hairong He, Yumei Zhao, Dongdong Niu, Jun Lyu

**Affiliations:** ^1^Clinical Research Center, The First Affiliated Hospital of Xi'an Jiaotong University, Xi'an, 710061 Shaanxi, China; ^2^School of Public Health, Xi'an Jiaotong University Health Science Center, Xi'an, 710061 Shaanxi, China; ^3^Clinical Research Service Center, The Affiliated Hospital of Guangdong Medical University, Zhanjiang, 524001 Guangdong, China; ^4^Department of Nephrology, The Affiliated Hospital of Guangdong Medical University, Zhanjiang, 524001 Guangdong, China; ^5^Department of Clinical Research, The First Affiliated Hospital of Ji'nan University, Guangzhou, 510632 Guangdong, China

## Abstract

**Background:**

Inflammation plays a key role in the pathophysiology and progression of acute kidney injury (AKI). Red cell distribution width (RDW) to platelet ratio (RPR) is a novel inflammatory index, and its prognostic effect on critically ill patients with AKI is rarely investigated. This work is aimed at investigating the association between RPR and in-hospital mortality in these patients.

**Methods:**

Data were extracted from the Medical Information Mart for Intensive Care III database. All-cause death during hospitalization was selected as the primary outcome. Receiver operating characteristic (ROC) curve was used to determine the optimal cut-off value, and the area under the curve (AUC) was applied to compare predictive ability among different indices. Cox proportional hazard models were utilized to assess the association between RPR and in-hospital mortality. Restricted cubic spline analysis for multivariate Cox model was performed to explore the shape of the relationship between RPR and mortality.

**Results:**

A total of 24,166 critically ill patients with AKI were included. The relationship of RPR and in-hospital mortality was nonlinear with a trend to rise rapidly and then gradually. For mortality prediction, RPR had the optimal cut-off value of 0.093, of which the AUC was 0.791 (95% confidence interval (CI): 0.773–0.810), which was higher than those of RDW, platelet, sequential organ failure assessment score, simplified acute physiology score II, neutrophil to lymphocyte ratio, and platelet to lymphocytes ratio. After adjustments for various confounders, high RPR showed a significant association with increased mortality with hazard ratios of 1.46 (95% CI: 1.40–1.55) for categorical variable and 1.88 (95% CI: 1.80–1.97) for continuous variables in the fully adjusted model.

**Conclusions:**

Elevated RPR on admission is substantially associated with high risk of in-hospital mortality in critically ill patients with AKI and thus may serve as a novel predictor of prognosis for these patients.

## 1. Introduction

Acute kidney injury (AKI) is a frequent complication in critically ill patients and has unacceptable morbidity and mortality. Its incidence rate varies between 30% and 70% in critically ill patients with an in-hospital mortality of 20–25% [1, 2]. In addition to short-term mortality, AKI is associated with the later development of chronic kidney disease, end-stage renal disease (ESRD), and long-term mortality [3]. Despite the extraordinary progress in therapies, the mortality rate of patients with AKI in the intensive care unit (ICU) has remained at a high level and even increased to 45%–60% when concomitant with other severe organ dysfunctions such as myocardial infarction or sepsis [4]. Identifying patients with AKI at high risk of death and providing them with timely and effective treatment can help improve the prognosis of this disease. Therefore, convenient and reliable tools for prognostic prediction must be developed to improve the management of critically ill patients.

Single- and multiparameter biomarkers (including anion gap, serum calcium, neutrophil-to-lymphocyte ratio (NLR), and platelet-to-lymphocyte ratio (PLR)) and scoring systems (including sequential organ failure assessment (SOFA), Acute Physiology and Chronic Health Evaluation II (APACHE II), and simplified acute physiology score II (SAPS II)) have been used in assessing the severity and prognosis of AKI in critically ill patients [5]. Unfortunately, most of these techniques are unsatisfactory due to low sensitivity or specificity. Complete blood count is a routine laboratory test in clinical practice that measures white blood cell (WBC), red blood cell (RBC), platelet count, and their morphological indices, such as red cell distribution width (RDW). RDW represents the heterogeneity of RBC volume in peripheral blood and has been widely used to differentiate the etiology of anemia. Its high value reflects the adverse outcomes of systematic inflammatory response, malnutrition, and bone marrow suppression [6]. For the past decade, RDW has gained substantial attention as an indicator of inflammation and is closely related to the prognosis of acute diseases, such as sepsis and AKI [7, 8]. Platelets are small bioactive masses of cytoplasm shed by cytoplasmic lysis of bone marrow megakaryocytes and play an important role in hemostasis. These cell fragments are key links in coagulation and inflammation, and thrombocytopenia is often regarded as a potential indicator of progressive inflammation during early stage of AKI [9]. Moreover, platelet count is significantly associated with prognosis in patients with AKI [10].

Changes in RDW and platelet count are complementary rather than isolated and serve as important components of hematological pathophysiology in the course of critical diseases. A new risk predictor, namely, RDW-to-platelet ratio (RPR), has been recently associated with the prognosis of various diseases, including neonatal sepsis [11], acute pancreatitis [12], and breast cancer [13]. Hence, RPR can reflect the status of systemic inflammatory severity in vivo. Given the key role of inflammation in the pathophysiology of AKI, RPR might be associated with the prognosis of AKI in critically ill patients. However, to the author's knowledge, only a few studies have explored the prognostic effect of RPR in AKI. Therefore, a retrospective cohort analysis was conducted to investigate the association between RPR and in-hospital mortality in critically ill patients with AKI.

## 2. Methods

### 2.1. Data Source

This was a data-mining study, of which data were obtained from the Medical Information Mart for Intensive Care III (MIMIC III) database [14, 15]. MIMIC III is an anonymous and publicly available database of more than 50,000 admission cases in various ICUs of the Beth Israel Deaconess Medical Center (BIDMC) in Boston, Massachusetts, from 2001 to 2012. Information in the MIMIC III database included demographics, vital signs, laboratory examinations, fluid balance, vital status, procedure codes, International Classification of Disease 9th revision (ICD-9) disease codes, medication, and nursing records. After the completion of a web-based course called “Protecting Human Research Participants,” the use of the MIMIC III database for research was approved by the institutional review boards of the Massachusetts Institute of Technology and BIDMC.

### 2.2. Participant Selection

Adult patients (≥18 years) diagnosed with AKI at first ICU admission during hospitalization were included. Kidney Disease: Improving Global Outcomes (KDIGO) criteria were applied for the diagnosis and classification of AKI [16]. Exclusion criteria were as follows: (1) no RDW or platelet measure on ICU admission, (2) stayed in ICU for less than 2 days, and (3) missing >10% individual data.

### 2.3. Data Extraction

The Structured Query Language with PostgreSQL (version 9.6) was used to extract data in the 24-hour admission from MIMIC III. The data included in this study was as follows: (1) demographic features, including age, sex, and ethnicity; (2) comorbidities, including congestive heart failure, chronic pulmonary, diabetes, and obesity; (3) laboratory examinations, including bicarbonate, bilirubin, chloride, glucose, hematocrit, hemoglobin, lactate, platelet, potassium, sodium, activated partial thromboplastin time (APPT), international normalized ratio (INR), WBC, lymphocyte, neutrophile, and RDW; (4) scoring systems, including sequential organ failure assessment (SOFA) and simplified acute physiology score II (SAPS II); (5) vital signs, including systolic blood pressure, diastolic blood pressure, temperature, heart rate, respiratory rate, and oxygen saturation (SpO_2_); (6) therapeutic and clinical management, including renal replacement therapy (RRT), mechanical ventilation, and vasopressor; and (7) renal function, including creatinine, blood urea nitrogen (BUN), and AKI stage. When the above indices had multiple results within 24 hours, the mean value was taken into analysis. The RPR was calculated by dividing the RDW (%) by the platelet count (10^9^/L). The NLR was calculated as the ratio of the neutrophil number to the lymphocyte number, while the PLR was calculated as the ratio of the platelet number to the lymphocyte number. The endpoint of this study was all-cause death during hospitalization in critically ill patients with AKI.

### 2.4. Statistical Analysis

Continuous variables were presented as median with interquartile range (IQR) and compared using the Wilcoxon rank-sum test. Categorical variables were presented as frequencies and proportion (%) and compared using the *χ*^2^ test. Hazard ratio (HR) and 95% confidence interval (CI) were calculated to predict the mortality risk by the Cox proportion risk model after adjustments for confounding factors. Receiver operating characteristic (ROC) curve was generated to calculate the area under the curve (AUC) and evaluate the discrimination of different parameters for mortality. ROC curve was also used to determine the optimal cut-off value by disclosing the trade-off between sensitivity and specificity. By using the optimal cut-off value of RPR, the patients were divided into two groups, namely, the high and low RPR groups, for further comparison. AUCs were compared by the *Z* test to ascertain differences in predictive performance for mortality among various indices. Restricted cubic spline analysis for the Cox model was performed with all covariates using RPR as continuous variable to explore the shape of the relationship between RPR and survival outcome. Subgroup analyses were also conducted to evaluate the effect of RPR on in-hospital mortality, including age, gender, ethnicity, congestive heart failure, chronic pulmonary, diabetes, obesity, renal replacement therapy, mechanical ventilation, vasopressor use, and AKI stage. All statistical analyses were performed by *R* software (version 4.0.5), and two-sided *P* values less than 0.05 were considered as statistically significant.

## 3. Results

### 3.1. Baseline Characteristics

Baseline characteristics of the included patients are listed in Table 1. A total of 24,166 patients, consisting of 13,897 (57.5%) men and 17,338 (71.7%) Caucasians with a median age of 68 years (IQR: 56-78), were included in this study. According to the optimal cut-off value of RPR (0.093) determined by ROC curve, 15,509 patients were in the low RPR group (<0.093), and the remaining 8657 were in the high RPR group (≥0.093). Patients in the high RPR group tend to be elderly; more men; and likely to have poor renal function, advanced AKI stage, and high SOFA and SAPS II scores. Patients in the high RPR group had high bilirubin, chloride, lactate, APTT, and INR and low glucose, hematocrit, hemoglobin, WBC, lymphocyte, and neutrophile. Moreover, they were more likely to receive RRT, mechanical ventilation, and vasopressor therapy.

During hospitalization, a total of 7805 patients (32.3%) suffered from death. The comparison of baseline characteristics in the surviving and nonsurviving patients is shown in Supplementary Table 1. Compared with the surviving patients, the nonsurviving patients were more likely to be older; were more men and Caucasian; have worse renal function, high disease severity score; and undergo RRT and vasopressor. The nonsurviving patients tend to accompany with congestive heart failure, chronic pulmonary, and diabetes. Moreover, the RPR level was much greater in the nonsurviving patients than the surviving patients.

### 3.2. Nonlinear Relationship between RPR and Mortality

**Figure 1:**
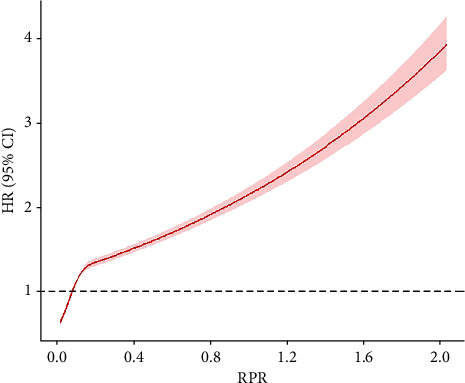
Association between RPR and in-hospital mortality in critically ill patients with acute kidney injury using multivariate-adjusted restricted cubic spline plot. A nonlinear relationship between RPR and in-hospital mortality was found with a trend to rise rapidly and then gradually, that is, a high RPR level indicates a high mortality risk. The range area indicates 95% confidence intervals. HR: hazard ratio; CI: confidence interval; RPR: red cell distribution width to platelet ratio.

The relationship between RPR and in-hospital mortality based on the restricted cubic spline analysis for the Cox model is shown in Figure 1. RPR and mortality exhibited a nonlinear relationship with a trend to rise rapidly and then gradually, that is, a high RPR level indicated a high mortality risk. Taking the value of 0.158 as the turning point, the slope of the low RPR part (<0.158) was steeper than that of the high RPR part (≥0.158).

### 3.3. Predictive Value of RPR Was Superior to Those of Other Indicators

**Figure 2:**
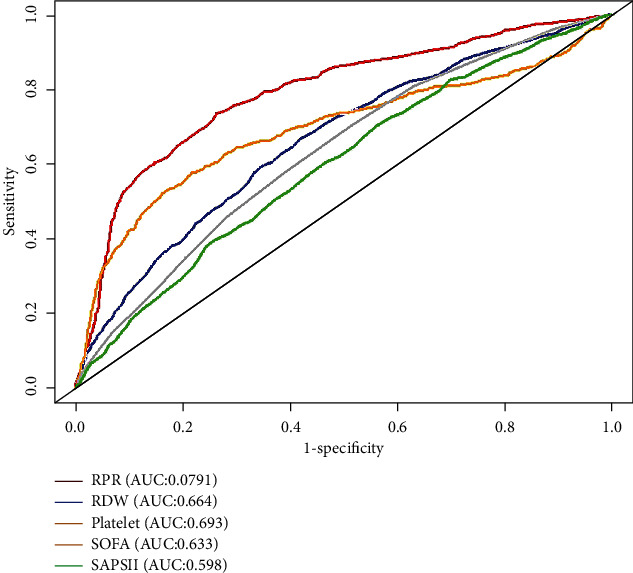
Receiver operating characteristics curves of RPR, RDW, platelet, SOFA, SAPS II score, NLR, and PLR for predicting in-hospital mortality in critically ill patients with acute kidney injury. The predictive ability of PRR for in-hospital mortality outperformed other indices, including RDW, platelet, SOFA score, SAPS II score, NLR, and PLR by comparing the area under the curve. RPR: red cell distribution width to platelet ratio; RDW: red cell distribution width; SOFA: sequential organ failure assessment; SAPS II: simplified acute physiology score II; NLR: neutrophil to lymphocyte ratio; PLR: platelet to lymphocyte ratio.

ROC analysis was applied to assess the predictive ability of RPR, RDW, platelet, SOFA, SAPS II, NLR, and PLR for in-hospital mortality (Figure 2). The optimal cut-off value of RPR for predicting mortality was 0.093 with a sensitivity of 0.734 and a specificity of 0.739 (Table 2). Its AUC was 0.791 (0.773–0.810), which was significantly higher than that of RDW at 0.664 (CI: 0.642–0.686) (*Z* = 8.936, *P* < 0.001), platelet at 0.693 (95% CI: 0.669–0.717) (*Z* = 6.533, *P* < 0.001), SOFA score at 0.633 (95% CI: 0.611–0.654) (*Z* = 11.117, *P* < 0.001), SAPS II score at 0.598 (95% CI: 0.576–0.620) (*Z* = 13.579, *P* < 0.001), NLR at 0.612 (95% CI: 0.590-0.634) (*Z* = 12.594, *P* < 0.001), and PLR at 0.617 (95% CI: 0.595-0.640) (*Z* = 12.242, *P* < 0.001) (Table 2). Among all indicators, the RPR had the best sensitivity and the second-best specificity for discriminating the patients at high risk of death.

### 3.4. Elevated RPR Was Significantly Associated with High Mortality

**Figure 3:**
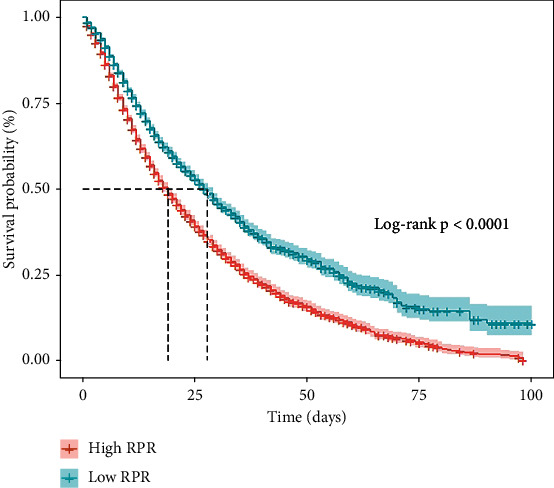
Kaplan-Meier analysis of in-hospital mortality by the RPR levels in critically ill patients with acute kidney injury. The in-hospital mortality rate in the high RPR group was significantly higher than that in the low RPR group (*P* for log-rank test < 0.001). RPR: red cell distribution width to platelet ratio.

The Kaplan-Meier analysis of in-hospital mortality by the RPR levels is shown in Figure 3. The in-hospital mortality rate in the high RPR group was significantly higher than that in the low RPR group (*P* for log-rank test < 0.001).

Cox regression analysis was used to further analyze the relationship between RPR and mortality. A crude model of univariate analysis revealed that a high RPR was significantly associated with a high mortality risk with HRs of 1.48 (1.42–1.55) for categorical variable and 2.05 (1.97–2.13) for continuous variable (Table 3, crude model). Multivariate analysis suggested that elevated levels of RPR, both expressed as categorical and continuous variables, are significantly correlated with an increasing risk of death during hospitalization, after adjustment for demographic features, comorbidities, laboratory examinations, scoring systems, clinical therapies, vital signs, and renal function (Table 3, Models 1–6). In the fully adjusted model, the HRs (95% CIs) of RPR for mortality risk were 1.46 (1.40–1.55) and 1.88 (1.80–1.97) when RPR was analyzed as categorical and continuous variables, respectively (Table 3, Model 6).

### 3.5. Prognostic Effects of RPR Based on Subgroup Analyses

A significant association between RPR and in-hospital mortality was observed in all subgroups (Table 4). Significant interactions were observed among congestive heart failure (*P* < 0.001), chronic pulmonary (*P* = 0.030), RRT (*P* = 0.019), and vasopressor use (*P* = 0.001). Patients with a high RPR who did not receive RRT were more likely to die (HR: 1.49, 95% CI: 1.42–1.56) than those receiving RRT (HR: 1.25, 95% CI: 1.08–1.46). A similar trend was observed in patients without congestive heart failure (HR: 1.70, 95% CI: 1.60–1.80), chronic pulmonary (HR: 1.54, 95% CI: 1.46–1.62), and vasopressor use (HR: 1.64, 95% CI: 1.54–1.75).

## 4. Discussion

The present study revealed that the relationship between RPR on ICU admission and all-cause in-hospital mortality was nonlinear with a trend to rise rapidly and then gradually. After adjustment of various confounders, elevated RPR was significantly associated with increased mortality. The predictive performance of RPR outperformed those of RDW, platelet, SOFA score, SAPS II score, NLR, and PLR.

Critically ill patients with AKI admitted in the ICU often have a poor clinical outcome, including prolonged hospitalization, dialysis requirement, CKD or ESED development, and death. Critically ill patients in the ICUs are dying of AKI and not just simply with AKI [17]. Even a slight change in serum creatinine levels is related to an increased risk of mortality. AKI develops as a result of a complex interaction between renal dysfunction and subsequent inflammation and coagulation [18]. As a marker of inflammation, RDW provides additional information for the prognosis of AKI [8, 19]. Jia et al. [8] reported that RDW outperforms traditional severity scoring systems in predicting the long-term prognosis of critically ill patients with AKI. However, the mechanisms underlying the relationship between high RDW and poor prognosis remain poorly understood. Cumulative evidence suggests inflammation, oxidative stress, and malnutrition as possible mechanisms. Inflammatory cytokines inhibit iron metabolism and bone marrow function and thus suppress erythropoietin production, resulting in RBC synthesis disorder and increased RDW values [20]. Oxidative stress associated with AKI, including disturbed metabolism, sepsis, and hemodynamic dysregulations, also increases the rate of RBC destruction, the release of immature erythrocytes into circulation, and consequently the RDW values [21]. Poor nutrition status, which is common in critically ill patients, can also lead to abnormal erythrocyte production and increased RDW levels [22].

Platelets act as a bridge between innate and acquired immune responses and contribute to the initiation or exacerbation of the inflammatory process by secreting proinflammatory cytokines and interacting with various kinds of immune cells, including neutrophils, T-lymphocytes, natural killer cells, and macrophages [23, 24]. A low platelet count represents progressive inflammation during the early stage of a disease and has been associated with an increased AKI risk induced by hemorrhagic shock and coronary artery bypass grafting surgery [25, 26]. Thrombocytopenia is common among critically ill patients and is significantly associated with poor prognosis [27]. Cho et al. [28] found that platelet count is an independent predictor for the all-cause mortality in patients with sepsis-induced AKI.

Based on these findings, the changes in RDW and platelet reflect the severity of inflammatory response and organ damage and thus are significantly associated with the prognosis of critically ill patients. By incorporating RDW and platelet, RPR has been recognized as a strong indicator of systematic inflammatory response in various diseases [29, 30]. The elevated levels of some inflammatory markers, such as C-reactive protein [31], PLR [32], NLR [33], and presepsin [34], are related to worse survival in critically ill patients with AKI. Therefore, it is biologically feasible that RPR is a reliable prognostic indicator in these patients. In this study, a close association was found between elevated RPR and poor in-hospital mortality, regardless of adjustments on various confounders. Moreover, the predictive performance of RPR outperformed NLR and PLR, indicating that the clinical utility of RPR may be superior to these traditional inflammatory indices. Despite progress in theoretical research, medical evidence and potential mechanisms on the association between RPR and prognosis in critically ill patients with AKI remain insufficient. Further studies are warranted to confirm the advantage of this parameter.

Some severity scoring systems, including SOFA and SAPS II scores, have been widely used to predict prognosis in critically ill patients. However, their application is controversial due to their poor predictive performance. Uchino et al. [35] found that neither SOFA nor SAPS II scores have adequate discrimination or calibration to predict mortality in patients with acute renal failure. Owing to the poor performance of these scores in patients with high risk, a multicentric study with 36,000 patients did not support their usage as a mortality prediction in ICU patients who underwent cardiac surgery [36]. RPR has advantages over these traditional scores in discrimination and predictive accuracy. In addition, SOFA and SAPS II scores are complex and time-consuming and therefore may be unsuitable for mortality prediction. By contrast, RDW and platelet, the main components of RPR, can be routinely tested with small amount of blood sample. This process is convenient, is cost-effective, requires easy calculation, and can be carried out in most hospitals. Thus, RPR might be more applicable to prognostic prediction than these scoring systems.

Significant associations were observed in all subgroups, suggesting the robustness of the results. Moreover, the prognostic effects of RPR were much higher in the subgroups of noncongestive heart failure, nonchronic pulmonary, nonvasopressor use, and non-RRT, indicating a possible interaction between RPR and these risk factors. Therefore, patients with AKI without congestive heart failure, chronic pulmonary, vasopressor use, and RRT would benefit most from RPR evaluation for clinical decision making. To the author's knowledge, this study is the first to explore these interactions. However, their underlying mechanism remains unclear. Critically ill patients with AKI who suffer from congestive heart failure or chronic pulmonary, use vasopressor, and receive RRT often had a poor disease severity, resulting in a relatively small difference in survival outcome among patients with different RPR levels.

Some limitations of this study should be acknowledged. First, given its single-center retrospective design, selection bias cannot be ignored, and the conclusions cannot be generalized to other centers. Further multicentric studies are needed to validate the results. Second, only RPR at admission was selected as an observation index, and the effect of dynamic RPR change on prognosis was not evaluated. Third, the factors influencing RDW, platelet, and RPR levels, such as iron deficiency anemia, vitamin B12 deficiency, malignancies, and immunological diseases, were not examined. Fourth, due to the missing items of past medical histories in the MIMIC III database, the RPR levels of patients before admission were unknown. Whether they had been treated with erythrocyte or platelet-raising drugs that might affect RPR values were also not specified. Finally, given that a high RPR is associated with adverse outcomes in various diseases, this study failed to answer whether interventions aimed at changing the RPR values may improve prognosis.

## 5. Conclusion

Elevated RPR upon admission is substantially associated with high risk of all-cause in-hospital mortality in critically ill patients with AKI. Therefore, the RPR may serve as a novel and convenient predictor of prognosis and the incorporation of PRR into routine assessment may facilitate the clinical decision making in these patients. However, the results are not conclusive and should be validated by further mple sizes.

## Figures and Tables

**Table 1:** Characteristics of the included patients.

Variables	All patients (*n* = 24166)	RPR		*P* value
		<0.093 (*n* = 15509)	≥0.093 (*n* = 8657)	
Demographic features				
Age (years)	68 (56-78)	67 (55-77)	68 (57-78)	<0.001
Male, *n* (%)	13897 (57.5)	8745 (56.4)	5152 (59.5)	<0.001
Caucasian ethnicity, *n* (%)	17338 (71.7)	11163 (72.0)	6175 (71.3)	0.283
Comorbidities				
Congestive heart failure, *n* (%)	7823 (32.4)	4956 (32.0)	2867 (33.1)	0.064
Chronic pulmonary, *n* (%)	5759 (23.8)	3819 (24.6)	1940 (22.4)	<0.001
Diabetes, *n* (%)	7587 (31.4)	5012 (32.3)	2575 (29.7)	<0.001
Obesity, *n* (%)	1776 (7.3)	1303 (8.4)	473 (5.5)	<0.001
Laboratory parameters				
Bicarbonate (mmol/L)	23.5 (21-26)	24 (21.5-26.5)	23.5 (20-25.5)	0.061
Bilirubin (mg/dL)	0.6 (0.4-1.15)	0.55 (0.4-0.9)	0.9 (0.5-2.0)	<0.001
Chloride (mmol/L)	105 (101.5-108)	105 (101.5-108)	106.5 (101.5-109)	<0.001
Glucose (mg/dL)	136.5 (116-165.5)	137.5 (117.5-167)	134.5 (114-163)	<0.001
Hematocrit (%)	32 (28.6-36)	33 (29.5-36.9)	30.4 (27.5-34)	<0.001
Hemoglobin (g/dL)	10.7 (9.55-12.1)	11.05 (9.8-12.5)	10.2 (9.2-11.4)	<0.001
Lactate (mmol/L)	1.85 (1.35-2.65)	1.8 (1.3-2.5)	2.0 (1.4-3.0)	<0.001
Potassium (mmol/L)	4.2 (3.85-4.65)	4.2 (3.85-4.6)	4.2 (3.9-4.7)	0.128
Sodium (mmol/L)	138.5 (136-140.5)	138.5 (136-140.5)	138 (136-140.5)	0.294
WBC (10^9^/L)	11.2 (8.35-14.9)	11.75 (9.1-15.5)	10 (7.1-13.9)	<0.001
Lymphocyte (%)	11.5 (6.9-18.4)	11.7 (7-18.5)	11.2 (6.3-18.4)	<0.001
Neutrophile (%)	80 (71.1-86.9)	80.5 (71.9-87)	79.3 (70-86.6)	<0.001
APTT (seconds)	32.2 (27.3-42)	30.6 (26.4-39)	35.5 (29.5-46.5)	<0.001
INR (seconds)	1.3 (1.15-1.6)	1.25 (1.1-1.45)	1.4 (1.2-1.75)	<0.001
RDW (%)	14.8 (13.8-16.4)	14.4 (13.5-15.7)	15.7 (14.4-17.5)	<0.001
Platelet (10^9^/L)	200 (146-267.5)	234.5 (195-288)	129 (100-153.5)	<0.001
NLR	7.5 (4.2-12.5)	6.2 (3.0-10.8)	10.2 (5.8-15.5)	<0.001
PLR	159 (39.5-211)	132.4 (24.5-182)	191 (43.7-255)	<0.001
Scoring systems				
SOFA	4 (2-7)	4 (2-5)	6 (4-8)	<0.001
SAPS II	36 (24-46)	34 (27-44)	40 (32-50)	<0.001
Therapies				
Renal replacement therapy, *n* (%)	1310 (5.4)	637 (4.1)	673 (7.8)	<0.001
Mechanical ventilation, *n* (%)	13457 (55.7)	8339 (53.8)	5118 (59.1)	<0.001
Vasopressor use, *n* (%)	10532 (55.7)	6186 (39.9)	4346 (50.2)	<0.001
Vital signs				
SBP (mmHg)	115 (106-128)	116 (107-129)	113 (105-125)	<0.001
DBP (mmHg)	59 (53-66)	59 (53-66)	58 (52-64)	<0.001
Temperature (°C)	36.8 (36.4-37.2)	36.8 (36.5-37.2)	36.8 (36.4-37.2)	0.521
Respiratory rate (beats/min)	18 (16-21)	18 (16-21)	18 (16-21)	0.779
Heart rate (beats/min)	85 (75-96)	85 (75-95)	85 (75-96)	0.505
SpO_2_ (%)	97.6 (96.2-98.7)	97.5 (96.1-98.7)	97.6 (96.2-98.8)	0.071
Renal function				
Creatinine (mg/dL)	1.05 (0.8-1.6)	1.0 (0.75-1.45)	1.15 (0.8-1.95)	<0.001
BUN (mg/dL)	20.5 (14.5-33.5)	19 (13.5-30)	23.5 (16-39.5)	<0.001
AKI stage, *n* (%)				<0.001
Stage 1	7010 (29.0)	4664 (30.1)	2346 (27.1)	
Stage 2	12015 (49.7)	8065 (52.0)	3950 (45.6)	
Stage 3	5141 (21.3)	2780 (17.9)	2361 (27.3)	

APTT: activated partial thromboplastin time; AKI: acute kidney injury; BUN: blood urea nitrogen; DBP: diastolic blood pressure; INR: international normalized ratio; NLR: neutrophil to lymphocyte ratio; PLR: platelet to lymphocyte ratio; RDW: red cell distribution width; RPR: red cell distribution width to platelet ratio; SAPS II: simplified acute physiology score II; SBP: systolic blood pressure; SOFA: sequential organ failure assessment; SpO_2_: oxygen saturation; WBC: white blood cell.

**Table 2:** Performance of red cell distribution width to platelet ratio for predicting in-hospital mortality in critically ill patients with acute kidney injury.

Variable	Cut-off	AUC (95% CI)	Sensitivity	Specificity
RPR	0.093	0.791 (0.773-0.810)	0.734	0.739
RDW	15.0	0.664 (0.642-0.686)	0.667	0.581
Platelet	142.9	0.693 (0.669-0.717)	0.530	0.830
SOFA	6.0	0.633 (0.611-0.654)	0.704	0.487
SAPS II	47.0	0.598 (0.576-0.620)	0.704	0.440
NLR	5.4	0.612 (0.590-0.634)	0.505	0.661
PLR	136	0.617 (0.595-0.640)	0.576	0.592

AUC: area under the curve; CI: confidence interval; RPR: red cell distribution width to platelet ratio; RDW: red cell distribution width; SOFA: sequential organ failure assessment; SAPS II: simplified acute physiology score II; NLR: neutrophil to lymphocyte ratio; PLR: platelet to lymphocyte ratio.

**Table 3:** Risk of in-hospital mortality in critically ill patients with acute kidney injury according to red cell distribution width to platelet ratio.

Model	Categorical variable (≥0.093 vs. <0.093)		Continuous variables	
	HR (95% CI)	*P* value	HR (95% CI)	*P* value
Crude model	1.48 (1.42-1.55)	<0.001	2.05 (1.97-2.13)	<0.001
Model 1^a^	1.48 (1.41-1.54)	<0.001	1.99 (1.91-2.07)	<0.001
Model 2^b^	1.48 (1.41-1.54)	<0.001	1.98 (1.90-2.06)	<0.001
Model 3^c^	1.49 (1.42-1.56)	<0.001	1.95 (1.88-2.02)	<0.001
Model 4^d^	1.48 (1.42-1.56)	<0.001	1.94 (1.88-2.01)	<0.001
Model 5^e^	1.47 (1.41-1.55)	<0.001	1.91 (1.84-1.99)	<0.001
Model 6^f^	1.46 (1.40-1.55)	<0.001	1.88 (1.80-1.97)	<0.001

HR: hazard ratio; CI: confidence interval. ^a^Model 1 was adjusted for demographic features, including age, gender, and ethnicity; ^b^Model 2 was additionally adjusted for comorbidities, including congestive heart failure, chronic pulmonary, diabetes, and obesity; ^c^Model 3 was additionally adjusted for laboratory examinations, including bicarbonate, bilirubin, chloride, glucose, hematocrit, hemoglobin, lactate, potassium, sodium, white blood cell, lymphocyte, neutrophile, activated partial prothrombin time, international normalized ratio, neutrophil to lymphocyte ratio, and platelet to lymphocyte ratio; ^d^Model 4 was additionally adjusted for scoring systems and clinical therapies, including sequential organ failure assessment, simplified acute physiology score II, renal replacement therapy, mechanical ventilation, and vasopressor use; ^e^Model 5 was additionally adjusted for vital signs, including systolic blood pressure, diastolic blood pressure, temperature, heart rate, respiratory rate, and SpO_2_; ^f^Model 6 was additionally adjusted for renal function, including creatinine, blood urea nitrogen, and AKI stage.

**Table 4:** Subgroup analyses of the association between red cell distribution width to platelet ratio and in-hospital mortality in critically ill patients with acute kidney injury.

Variables	*N*	HR (95% CI)	*P* value	*P* for interaction
Age				0.075
≤65 years	10895	2.08 (1.93-2.25)	<0.001	
>65 years	13271	1.24 (1.18-1.32)	<0.001	
Gender				0.375
Male	13897	1.46 (1.38-1.55)	<0.001	
Female	10269	1.52 (1.42-1.63)	<0.001	
Ethnicity				0.803
Caucasian	17338	1.49 (1.41-1.157)	<0.001	
Others	6828	1.49 (1.36-1.62)	<0.001	
Congestive heart failure				<0.001
No	16343	1.70 (1.60-1.80)	<0.001	
Yes	7823	1.24 (1.16-1.33)	<0.001	
Chronic pulmonary				0.030
No	18407	1.54 (1.46-1.62)	<0.001	
Yes	5759	1.35 (1.24-1.47)	<0.001	
Diabetes				0.407
No	16579	1.52 (1.44-1.60)	<0.001	
Yes	7587	1.45 (1.34-1.57)	<0.001	
Obesity				0.455
No	22390	1.46 (1.40-1.53)	<0.001	
Yes	1776	1.52 (1.23-1.88)	<0.001	
Renal replacement therapy				0.019
No	22856	1.49 (1.42-1.56)	<0.001	
Yes	1310	1.25 (1.08-1.46)	0.004	
Mechanical ventilation				0.084
No	10709	1.55 (1.45-1.65)	<0.001	
Yes	13457	1.44 (1.35-1.53)	<0.001	
Vasopressor use				0.001
No	13634	1.64 (1.54-1.75)	<0.001	
Yes	10532	1.35 (1.27-1.44)	<0.001	
AKI stage				0.233
Stage 1	7010	1.57 (1.43-1.72)	<0.001	
Stage 2	12015	1.44 (1.34-1.54)	<0.001	
Stage 3	5141	1.36 (1.26-1.48)	<0.001	

HR: hazard ratio; CI: confidence interval; AKI: acute kidney injury.

## Data Availability

The data used to support the findings of this study were supplied by the Medical Information Mart for Intensive Care III (MIMIC III) database.

## Supplementary Materials

Supplementary Materials Supplementary Table 1: comparison of baseline characteristics in the surviving and nonsurviving patients.
