# Supplementary material for: Red Cell Distribution Width to Platelet Ratio Is Associated with Increasing In-Hospital Mortality in Critically Ill Patients with Acute Kidney Injury
Source: Dis Markers. 2022 Jan 17;2022:4802702. doi: 10.1155/2022/4802702 (PMC8786548; doi:10.1155/2022/4802702)
Supplement: Supplementary Materials — Supplementary Table 1: comparison of baseline characteristics in the surviving and nonsurviving patients. [file 4802702.f1.pdf]

Supplementary Table 1. Comparison of baseline characteristics in the surviving and non-surviving patients.

Variables	All patients (n = 24166)	Surviving patients (n = 16361)	Non-surviving patients (n = 7805)	*P* value
Demographic features				
Age (years)	68 (56-78)	66 (54-76)	71 (60-80)	< 0.001
Male, n (%)	13897 (57.5)	9510 (58.1)	4387 (56.2)	0.005
Caucasian ethnicity, n (%)	17338 (71.7)	11647 (71.2)	5691 (72.9)	0.005
Comorbidities				
Congestive heart failure, n (%)	7823 (32.4)	4554 (27.8)	3269 (41.9)	< 0.001
Chronic pulmonary, n (%)	5759 (23.8)	3699 (22.6)	2060 (26.4)	< 0.001
Diabetes, n (%)	7587 (31.4)	5002 (30.6)	2585 (33.1)	< 0.001
Obesity, n (%)	1776 (7.3)	1422 (8.7)	354 (4.5)	< 0.001
Laboratory parameters				
Bicarbonate (mmol/L)	23.5 (21-26)	24 (22.0-26.0)	23.0 (20.0-25.0)	< 0.001
Bilirubin (mg/dl)	0.6 (0.4-1.15)	0.6 (0.4-1.05)	0.7 (0.4-1.5)	< 0.001
Chloride (mmol/L)	105 (101.5-108)	105.5 (102-108.5)	104 (100-108)	< 0.001
Glucose (mg/dl)	136.5 (116-165.5)	135.5 (117-161.5)	139 (113.5-175.5)	< 0.001
Hematocrit (%)	32 (28.6-36)	32.5 (29-36.5)	31.1 (28-35)	< 0.001
Hemoglobin (g/dl)	10.7 (9.55-12.1)	10.9 (9.7-12.3)	10.3 (9.3-11.6)	< 0.001
Lactate (mmol/L)	1.85 (1.35-2.65)	1.8 (1.3-2.55)	2.0 (1.4-3.1)	< 0.001
Potassium (mmol/L)	4.2 (3.85-4.65)	4.2 (3.85-4.6)	4.25 (3.85-4.7)	< 0.001
Sodium (mmol/L)	138.5 (136-140.5)	138.5 (136-140.5)	138 (136.5-141)	0.983
WBC (10^9/L)	11.2 (8.35-14.9)	11.15 (8.5-14.5)	11.35 (8.1-15.7)	0.118
Lymphocyte, (%)	11.5 (6.9-18.4)	12.4 (7.5-19.4)	10 (5.6-16)	< 0.001
Neutrophile, (%)	80 (71.1-86.9)	79.4 (70.7-86.1)	81.5 (72.6-88)	< 0.001
APTT (seconds)	32.2 (27.3-42)	31.5 (26.9-40)	34.2 (28.1-46.9)	< 0.001
INR (seconds)	1.3 (1.15-1.6)	1.3 (1.15-1. 5)	1.4 (1.2-1.8)	< 0.001
RDW (%)	14.8 (13.8-16.4)	14.4 (13.6-15.7)	15.8 (14.5-17.5)	< 0.001
Platelet (10^9/L)	200 (146-267.5)	200.5 (150.5-264)	198.5 (134-276)	0.330
NLR	7.5 (4.2-12.5)	6.7 (3.7-11.6)	9 (5.3-14.8)	< 0.001
PLR	159 (39.5-211)	137 (25.5-188)	186 (40.2-263)	< 0.001
Scoring systems				
SOFA	4 (2-7)	4 (2-6)	5 (3-8)	< 0.001
SAPS II	36 (24-46)	34 (26-42)	43 (35-54)	< 0.001
Therapies				
Renal replacement therapy, n (%)	1310 (5.4)	623 (3.8)	687 (8.8)	< 0.001
Mechanical ventilation, n (%)	13457 (55.7)	9373 (57.3)	4084 (52.3)	< 0.001
Vasopressor use, n (%)	10532 (55.7)	6822 (41.7)	3710 (47.5)	< 0.001
Vital signs				
SBP (mmHg)	115 (106-128)	116 (107-128)	113 (103-127)	< 0.001
DBP (mmHg)	59 (53-66)	59 (54-66)	57 (51-65)	< 0.001
Temperature (°C)	36.8 (36.4-37.2)	36.9 (36.5-37.2)	36.7 (36.3-37.2)	< 0.001
Respiratory rate (beats/min)	18 (16-21)	18 (16-20)	19 (17-23)	< 0.001

Heart rate (beats/min)	85 (75-96)	84 (75-95)	86 (75-99)	< 0.001
SpO_2 (%)	97.6 (96.2-98.7)	97.7 (96.3-98.7)	97.4 (95.8-98.7)	< 0.001
Renal function				
Creatinine, (mg/dl)	1.05 (0.8-1.6)	0.95 (0.75-1.35)	1.3 (0.9-2.3)	< 0.001
BUN, (mg/dl)	20.5 (14.5-33.5)	18 (13-27.5)	28 (18.5-46)	< 0.001
AKI stage, n (%)				< 0.001
Stage 1	7010 (29.0)	5178 (31.6)	1832 (23.5)	
Stage 2	12015 (49.7)	8626 (52.7)	3389 (43.4)	
Stage 3	5141 (21.3)	2557 (15.6)	2584 (33.1)	
RPR	0.077 (0.058-0.112)	0.072 (0.055-0.098)	0.094 (0.066-0.182)	< 0.001

APTT, activated partial thromboplastin time; AKI, acute kidney injury; BUN, blood urea nitrogen; DBP, diastolic blood pressure; INR, International Normalized Ratio; NLR, neutrophil to lymphocyte ratio; PLR, platelet to lymphocyte ratio; RDW, red cell distribution width; RPR, red cell distribution width to platelet ratio; SAPS II, simplified acute physiology score II; SBP, systolic blood pressure; SOFA, sequential organ failure assessment; SpO2, oxygen saturation; WBC, white blood cell.
